# Effects of Intravenous Administration of Human Umbilical Cord Blood Stem Cells in 3-Acetylpyridine-Lesioned Rats

**DOI:** 10.1155/2012/135187

**Published:** 2012-10-24

**Authors:** Lucía Calatrava-Ferreras, Rafael Gonzalo-Gobernado, Antonio S. Herranz, Diana Reimers, Teresa Montero Vega, Adriano Jiménez-Escrig, Luis Alberto Richart López, Eulalia Bazán

**Affiliations:** ^1^Servicio de Neurobiología, Instituto Ramón y Cajal de Investigación Sanitaria (IRYCIS), 28034 Madrid, Spain; ^2^Servicio de Bioquímica, Instituto Ramón y Cajal de Investigación Sanitaria (IRYCIS), 28034 Madrid, Spain; ^3^Servicio de Neurología, Hospital Universitario Ramón y Cajal, 28034 Madrid, Spain; ^4^Centro de Transfusiones de la Comunidad de Madrid, Valdebernardo, 28030 Madrid, Spain; ^5^Servicio de Neurobiología-Investigación, Hospital Ramón y Cajal, Carretera de Colmenar Km. 9, 1, 28034 Madrid, Spain

## Abstract

Cerebellar ataxias include a heterogeneous group of infrequent diseases characterized by lack of motor coordination caused by disturbances in the cerebellum and its associated circuits. Current therapies are based on the use of drugs that correct some of the molecular processes involved in their pathogenesis. Although these treatments yielded promising results, there is not yet an effective therapy for these diseases. Cell replacement strategies using human umbilical cord blood mononuclear cells (HuUCBMCs) have emerged as a promising approach for restoration of function in neurodegenerative diseases. The aim of this work was to investigate the potential therapeutic activity of HuUCBMCs in the 3-acetylpyridine (3-AP) rat model of cerebellar ataxia. Intravenous administered HuUCBMCs reached the cerebellum and brain stem of 3-AP ataxic rats. Grafted cells reduced 3-AP-induced neuronal loss promoted the activation of microglia in the brain stem, and prevented the overexpression of GFAP elicited by 3-AP in the cerebellum. In addition, HuUCBMCs upregulated the expression of proteins that are critical for cell survival, such as phospho-Akt and Bcl-2, in the cerebellum and brain stem of 3-AP ataxic rats. As all these effects were accompanied by a temporal but significant improvement in motor coordination, HuUCBMCs grafts can be considered as an effective cell replacement therapy for cerebellar disorders.

## 1. Introduction

Cerebellar ataxias (CAs) include a heterogeneous group of infrequent diseases characterized by lack of motor coordination [1]. According to their etiology, they can be divided into sporadic forms and hereditary diseases. All of them have in common cerebellum and associated neuronal circuits dysfunction, in particular spinocerebellar afferents [2–5]. Current therapeutic approaches are based on the use of drugs that correct some of the molecular processes involved in the pathogenesis of this group of diseases [1, 6–8]. Furthermore, other studies have assayed the potential therapeutic activity of intracerebroventricular, peripheral, or intranasal administration of neurotrophic factors such as insulin-like growth factor (IGF-I), or glial-derived growth factor (GDNF), in different experimental models of cerebellar ataxia in rodents [9–13]. Although the above-mentioned treatments (drugs and trophic factors) yielded promising results, there is not yet an effective therapy for these types of diseases to date [1].

Cell replacement strategies using stem cells (SCs) as donor tissue have emerged as a promising approach for restoration of function in neurodegenerative diseases [14–19]. Hematopoietic stem cells from human umbilical cord blood (HuUCBCs) have been proposed as an excellent source of embryonic SCs in regenerative therapies for the Central Nervous System [20–23]. HuUCBCs are easily accessible they retain certain properties of embryonic SCs such as the expression of transcription factors specific to embryonic antigens [24] and are well tolerated by the host due to their low immunogenicity [25]. Additionally, in vitro manipulation of HuUCBCs has shown their plasticity. Thus, after exposure to different agents, these cells are able to express antigens of diverse cellular lineages, including the neural type [26–31]. 

HuUCBCs were used successfully for the first time in 1989, as a bone marrow transplant in a patient with Fanconi's anemia [32]. Other studies have shown that systemic administration of HuUCBCs to different experimental models of neurodegenerative diseases improved their neurological symptoms and life expectancy [22, 23]. The beneficial effects of HuUCBCs seemed to be due to their ability to synthesize and release trophic factors involved in cell survival, rather than having a role in neuronal replacement [23, 33–36].

Stem cell-reparative approaches have been proposed for cerebellum-related disorders [37–40]. However, the type of stem cells most appropriate for future human cell therapy is not clearly defined at present [37]. Considering the possibility that HuUCBCs could be used as a therapeutic agent in CA, we analyze their potential neuroregenerative and/or neuroprotective activity in the 3-acetylpyridine (3-AP) experimental model of CA in rats. The rationale for using this CA model was because the neurotoxin 3-AP selectively lesions calbindin expressing neurons in the inferior olive [9], and this nucleus plays a key role in the control of the cerebellar function by sending glutamatergic excitatory signals to Purkinje cells [41, 42].

Here, we report that intravenous administration of HuUCB mononuclear cells (HuUCBMCs) reaches the cerebellum and brain stem of 3-AP-lesioned rats. Grafted cells reduce neuronal loss in the brain stem, prevent glial reactivity in the cerebellum, and improve motor coordination in ataxic rats. In this study, we also show that HuUCBMCs upregulate the expression of proteins that are critical for cell survival, such as phospho-Akt and Bcl-2, in the cerebellum and brain stem of 3-AP-lesioned rats. The role of activated microglia in HuUCBMCs-mediated neuronal protection in the brain stem is also discussed. 

## 2. Materials and Methods

### 2.1. Experimental Model of Cerebellar Ataxia in Rats

A total of 40 female Sprague Dawley rats weighing 220–250 g were used in accordance with the European Union Council Directive (86/609/EEC). Rats received an intraperitoneal (i.p.) injection of the neurotoxine 3-AP (40 mg/kg) that selectively lesioned calbindin expressing neurons in the inferior olive [9]. This nucleus plays a key role in the control of the cerebellar function by sending glutamatergic excitatory signals to Purkinje cells (PCs) [41, 42]. From a histological point of view, PC and granule neurons of the cerebellar cortex are the most commonly affected population of neurons in CA [3].

### 2.2. Behavioral Testing

Motor performance was analyzed using the rotarod test. Before 3-AP lesions were produced, rats received 9 independent training sessions in the rotarod (PanLab S.L., Mod. LE 8500, Cornellá, Spain), with 4 1-minute evaluations at 40 rpm (fixed speed), and 4 1-minute evaluations at 4 to 40 rpm (accelerating rod). Those animals that withstood more than 1 minute at 40 rpm and at 4 to 40 rpm were selected for 3-AP lesions. Motor coordination was evaluated at 72 hours after lesion. Those animals resulting in mean latencies to fall on the accelerating rod of approximately 19 ± 3 s (*n* = 16) were selected for HuUCBMCs or vehicle administration. Starting 10 days after 3-AP lesion procedure, animals were monitored once a week until the end of the study period.

### 2.3. HuUCBMCs Isolation of Blood Cell Concentrate

Assessment, processing, and cryopreservation of HuUCBCs were carried out by the Centro de Transfusiones de la Comunidad de Madrid (Valdebernardo) in accordance with the Spanish Directive for Donors' Selection (Edition 4/May 2009/PO.CO.01). The donated units met the criteria for minimal cellularity and volume showing the following parameters: mononuclear cells (MCs): 337.8 × 10^6^ ± 32.56, total nucleated cells (TNCs): 910.8 × 10^6^ ±  86.77, and cells positives for CD34: 0.7620 × 10^6^ ± 0.09140. 

For isolation of HuUCBMCs we followed a methodology previously described [43]. Briefly, HuUCBCs were drawn from the bag and divided into 2 Falcon tubes with half volume of Lymphoprep and centrifuged at 800 g and 20°C for 40 min to create a Ficoll gradient. The band of mononuclear cells located at the interface (2 to 7 mL) was taken and washed 3 times with PBS. An aliquot of 40 *μ*L was used to determine the number of living cells by Trypan Blue using a Neubahuer chamber.

### 2.4. Cell Transplantation

At 3 days after 3-AP lesion, rats were anesthesized by inhalatory administration of Isoflurane (2%). One group of animals (*n* = 14) received a single injection of 4.5 × 10^6^ HuUCBMNCs in 250 *μ*L of sterile PBS into the lateral vein of the tail (3-AP + cells). Another group of 3-AP-lesioned rats (*n* = 10) received the same volume of sterile PBS (3-AP + vehicle). As controls we used a group of naïve rats that did not receive HuUCBMNCs grafts (*n* = 6). All experimental groups received cyclosporine (5 mg/kg i.p.) once a day to avoid rejection of human cells 24 hours before cell transplantation, and until the end of the study. Animals were sacrificed at 1, 7, 21 and 44 days after transplantation (4, 10, 24, and 48 days after lesion, resp.).

### 2.5. Tissue Processing

At 4, 10, and 21 days after lesion the animals were perfused intracardially under deep anesthesia with 50 mL of isotonic saline, followed by 250 mL of 4% paraformaldehyde. Brains were postfixed in the same solution for 24 hr at 4°C, cryoprotected and frozen, before sectioning on a cryostat. For the inferior olive 20 *μ*m thick coronal sections were performed at three levels separated by a distance of approximately 400 *μ*m. These levels correspond to the following coordinates of the stereotaxic atlas of Paxinos and Watson [44]: −13.30 mm from Bregma (Zone 1), −12.80 mm (zone 2) and −11.96 mm (zone 3).

For immunohistochemical analysis of the cerebellum, 20 *μ*m thick coronal sections were obtained in the cryostat, and mounted on positively charged slides (Dako REAL Capillary Gap microscope slides).

### 2.6. Antibodies and Immunochemicals

The primary antibodies used in this study were rabbit antiproliferating cell nuclear antigen (PCNA, 1 : 75; Santa Cruz Biotechnology Inc., Santa Cruz, CA, USA), rabbit antiglial fibrillary acidic protein (GFAP, 1 : 200; DakoCytomation),  mouse antineuronal nuclei (NeuN, 1 : 1000; Chemicon International Inc.), mouse anti-OX6 (1 : 250; AbD Serotec, Oxford, UK), rabbit antilaminin (1 : 25; Sigma Chemical Co., St. Louis, MO, USA), mouse anti-Bcl-2 (1 : 25; Santa Cruz Biotech), rabbit anticalbindin (1 : 500; Millipore, Temecula, CA, USA), mouse anti-human leucocyte antigen ABC (HLA-ABC 1 : 500; AbD Serotec, Oxford, UK), mouse anti-human nuclei protein (HuNu, 1 : 25; Millipore, Temecula, CA, USA), and rabbit anti-Bax (1 : 250; Santa Cruz Biotech). The secondary antibodies and other immunochemicals used were peroxidase-labeled isolectin B4 (IB4, 1 : 20; Sigma Chemical Co, St. Louis, MO, USA), biotinylated goat anti-mouse IgG (Zymed Laboratories; South San Francisco, CA, USA), streptavidin-biotin-peroxidase complex (DakoCytomation), diaminobenzidine (DAB) + substrate-chromogen system (both from DakoCytomation), Alexa Fluor-568 goat anti-mouse IgG, and Alexa Fluor-488 goat anti-rabbit IgG (1 : 400; all from Molecular Probes; Eugene, OR, USA), fluorescein-conjugated goat anti-mouse IgG (1 : 25; Jackson ImmunoResearch Laboratories Inc., West Grove, PA, USA), Cy3-conjugated donkey anti-guinea pig IgG (1 : 500, Jackson ImmunoResearch Laboratories Inc.), and rhodamine-conjugated goat anti-rabbit IgG (1 : 100, Chemicon International Inc.). 

### 2.7. Immunohistochemistry and Morphometric Analysis

Tissue sections were treated with sodium acetate 10 mM, pH 6.0, at 95°C for 4 min, and preincubated with 5% normal goat serum (NGS) in Tris-buffered saline (TBS: 0.15 M NaCl and 0.1 M Tris HCl, pH 7.4)/0.1% Triton X-100 for 30 min. Primary antibodies were applied for 24 hr at 4°C, and most of them were visualized using immunofluorescence procedures. The slides were coverslipped in a medium containing p-phenylenediamine and bisbenzimide (Hoechst 33342; Sigma) for the detection of nuclei. Some series of sections were preincubated with 5% NGS in TBS and then processed for the histochemical detection of IB4, a marker of microglia and macrophages, by incubating for 2 hr with IB4 conjugated to peroxidase. Finally, the reaction product was detected with DAB chromogen. 

For quantitative estimation of calbindin immunostaining in the inferior olive, measurements were performed in several coronal sections of the brainstem, at the three levels indicated previously (see tissue processing). The area of the olive was demarcated and measured in each zone, and the number of cells with caldindin staining was assessed using the 20X objective. Immunohistochemical results were expressed as the number of positive cells/section with the aid of the Computer-Assisted Stereology Toolbox (CAST) grid system (Olympus, Ballerup, Denmark). Fluorescence images were acquired and analyzed by confocal microscopy (Nikon C1 plus ECLIPSE Ti-e microscope).

### 2.8. Western Blotting Protein Analysis

After 44 days of cells transplantation, the brain stem and cerebellum of 3-AP-lesioned rats that received vehicle (*n* = 8) or cells (*n* = 9) were removed and dissected following a previously described methodology [45]. Tissue was homogenized (1 : 8, w/v) with homogenization buffer (20 mM Tris-HCl, pH 7.5: 140 mM potassium chloride; 5 mM magnesium acetate; 1 mM dithiothreitol, 2 mM benzamidine, 1 mM EDTA, 2 mM EGTA, 0.5% Triton X-100, 10 *μ*g/mL pepstatin A, 10 *μ*g/mL leupeptin, and 10 *μ*g/mL antipain; 20 mM sodium *β*-glycerophosphate; 20 mM sodium molybdate; 200 mM sodium orthovanadate). Homogenates were centrifuged at 11,000 g for 20 min, and proteins were processed for Western blot analysis to determine the relative levels of several proteins. The procedures were performed at 4°C, and samples were kept at −80°C until use. Aliquots of 30 *μ*g of protein were separated by electrophoresis on 10–15% SDS-polyacrylamide minigels and transferred to nitrocellulose filters. Membranes were soaked in blocking solution (0.1 M PBS and 5% dry skimmed milk, pH 7.4) and incubated with the following primary antibodies diluted in 0.1 M PBS and 1% dry skimmed milk, pH 7.4: mouse anti-Bcl-2 (1 : 400; Santa Cruz Biotechnology Inc., Burlingame, CA, USA), rabbit anti-Bax (1 : 300; Santa Cruz Biotechnology, Santa Cruz, CA, USA), rabbit antiproliferating cell nuclear antigen (PCNA, 1 : 1000; Santa Cruz Biotechnology, Santa Cruz, CA, USA), rabbit antiglial fibrillary acidic protein (GFAP, 1 : 5000; DakoCytomation, Denmark), rabbit anti-Glut5 (1 : 500; Abcam), mouse anti-OX6 (1 : 1000, AbD Serotec, Oxford, UK), rabbit anti-HuNu protein (1 : 200, Millipore, Temecula, CA, USA), rabbit anticalbindin (1 : 5000; Millipore, Temecula, CA, USA), rabbit anti-Akt (Ser473P) (1 : 2000; Cell Signaling Technology, Beverly, MA, USA), rabbit anti-Akt (1 : 2000; Cell Signaling Technology). After extensive washing in 0.05% PBS-Tween, membranes were incubated with the peroxidase-conjugated or alkaline-phosphatase-conjugated secondary antibodies diluted 1 : 2000 in blocking solution. The membranes were developed with enhanced chemiluminescence Western blotting, following the manufacturer's instructions (Amersham, Buckinghamshire, England), and were exposed to hyperfilm. Membranes were also immunolabeled for loading control using mouse anti-*β* actin (1 : 5000; Sigma Aldrich) and anti-mouse IgG alkaline phosphatase-conjugated (1 : 3000, Sigma Aldrich) and were developed with alkaline phosphatase reagent. The density of stained bands was scanned and quantified with the Image QuantTL software package, and the data were normalized in relation to *β* actin levels.

### 2.9. Data Analysis

Results are expressed as mean ± SEM of (*n*) independent animals. Statistical analyses for immunohistochemical and biochemical studies were performed using one-way ANOVA followed by the Newman-Keuls multiple comparison test. For behavioral studies, a two-way ANOVA followed by Student's *t*-test was used. Differences were considered significant when *P* ≤ 0.05. 

## 3. Results

### 3.1. HuUCBMCs Grafts Ameliorate Motor Coordination in 3-AP-Lesioned Rats

**Figure 1:**
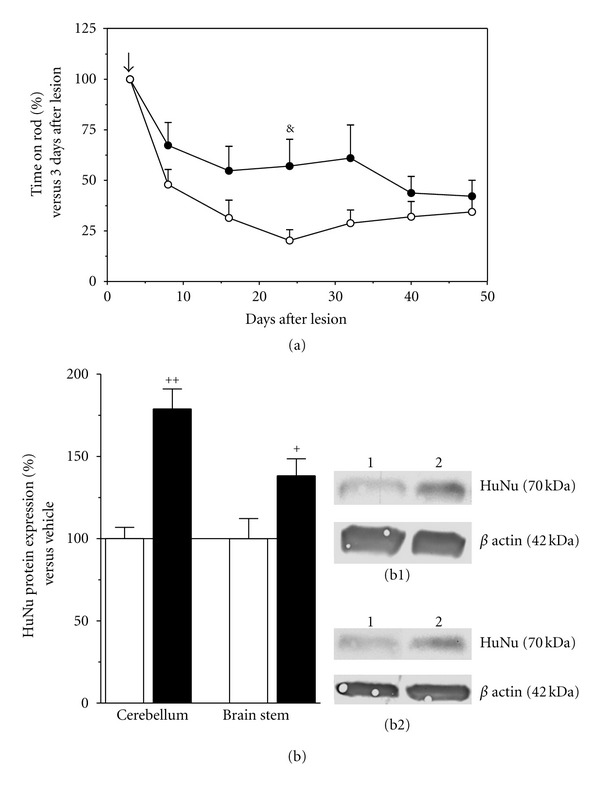
HuUCBMCs grafts improve motor coordination and reach the cerebellum and brain stem of 3-AP ataxic rats. Motor performance, as assessed by the rotarod test, shows a progressive impairment in 3-AP-lesioned rats receiving vehicle ((a) white circles) that reaches a plateau between 15 and 24 days after lesion. Motor coordination in 3-AP-lesioned rats that received HuUCBMCs grafts ((a) black circles) is significantly improved at 24 days after lesion. Time of implantation of HuUCBMCs (**↓**). (b) shows the detection by western blot of HuNu protein in the cerebellum and brain stem of 3-AP-lesioned rats treated with vehicle (white bars), or with HuUCBMCs (black bars). (b1) and (b2) show representative blots for HuNu protein in the cerebellum (b1) and brain stem (b2). Lane 1: 3-AP-lesioned rats treated with vehicle (3-AP + vehicle); lane 2: 3-AP-lesioned rats treated with HuUCBMCs grafts (3-AP + cells). Results represent the mean ± SEM of 4 (b) to 10 (a) individual animals. ^&^ *P* ≤ 0.05 versus 3-AP + vehicle rats at 24 days after lesion, ^+^ *P* ≤ 0.05, ^++^ *P* ≤ 0.01 versus 3-AP + vehicle rats at 48 days after lesion.

To determine whether HuUCBMCs transplantation was functional in vivo, we have analyzed motor coordination using the rotarod test. Motor performance of naïve rats was relatively stable over repeated tests, resulting in mean latencies to fall on the accelerating rod of approximately 52.31 ± 2.7 s (*n* = 16). At 3 days after 3-AP lesion, the latency to fall from rotarod was reduced to 19 ± 3 sec (*n* = 16). As shown in Figure 1(a), 3-AP-lesioned rats showed a progressive impairment that reached a plateau between 15 and 24 days after lesion. By contrast, in the 3-AP + cells group of animals, motor performance was stable between 8 and 32 weeks after lesion (Figure 1(a)). Moreover, 21 days after HuUCBMCs transplantation (24 days after lesion), their motor coordination was significantly improved, to compared with 3-AP-lesioned rats receiving vehicle (Figure 1(a)).

### 3.2. Detection of HuUCBMCs in Brain Stem and Cerebellum of 3-AP-Lesioned Rats

**Figure 2:**
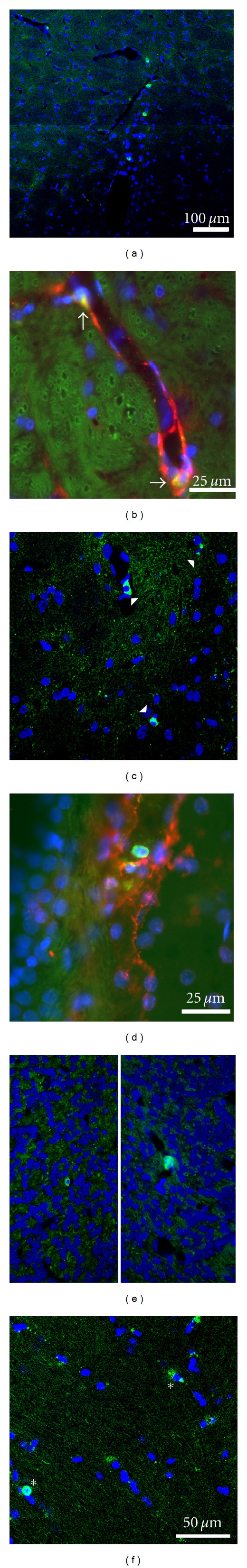
Immunodetection of human leucocyte antigen-ABC in the brain stem and cerebellum of 3-AP ataxic rats. (a) and (c) show HLA-ABC immunostaining (green) in the ventral (a) and dorsal (c) brain stem of 3-AP-lesioned rats receiving HuUCBMCs grafts. (b) Shows HLA-ABC (b, green) and laminin (b, red) immunoreactivity in the ventral brain stem. Note how HLA-ABC-positive cells are associated to laminin-positive blood vessels (b, yellow, white arrows) or integrated in the parenchyma (c, white arrowheads). In the cerebellum HLA-ABC-positive cells (d–f, green) are located near to the vermis in laminin-positive blood vessels (d, red), and in the parenchyma of the granular (e, green) and molecular layers (f, green, white stars) of the cerebellar cortex. Nuclei were counterstained with Hoechst 33342 (blue). Scale bar: 25 *μ*m (b and d), 50 *μ*m (c, e, and f), and 100 *μ*m (a).

Immunohistochemical analysis for the human endogenous marker HLA-ABC was performed to determine if intravenous transplanted HuUCBMCs were able to reach the brain stem and cerebellum of 3-AP-lesioned rats. Seven days after HuUCBMCs transplantation, 3-AP-lesioned rats showed HLA-ABC-positive cells in ventral (Figure 2(a)) and dorsal (Figure 2(c)) zones of the brain stem. These cells were associated to laminin-positive blood vessels (Figure 2(b)), or integrated in the parenchyma (Figure 2(c)). Similarly, the cerebellum of 3-AP + cells treated rats showed HLA-ABC immunoreactivity (Figures 2(d)–2(f)). Thus, HLA-ABC-positive cells were observed in the vermis associated to blood vessels (Figure 2(d)), and in the parenchyma of the granular (Figure 2(e)) and molecular (Figure 2(f)) layers of the cerebellar cortex. Under our experimental conditions, HLA-ABC immunoreactivity was not observed at longer periods after transplantation (i.e. 21 days). However, by western blot analysis we found that 45 days after HuUCBMCs grafts were performed (48 days after lesion), the cerebellum and brain stem of 3-AP-lesioned rats showed significant levels of the nuclear antigen expressed by human cells HuNu, compared to 3-AP + vehicle-treated rats, where HuNu protein expression was very low in both structures (Figure 1(b)). We were unable to confirm these results by immunohistochemistry because the antibody used for HuNu detection gave a high background in rat brain slices.

### 3.3. HuUCBMCs Grafts Partially Prevent Neurotoxin-Induced Neuronal Loss in the Brain Stem

**Figure 3:**
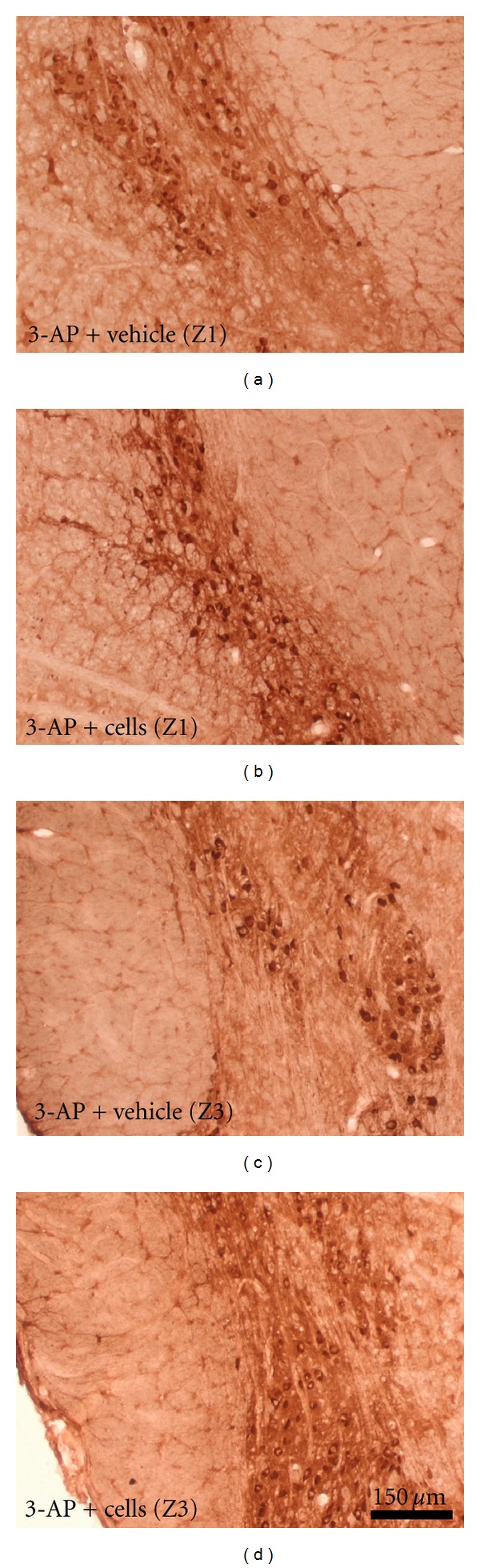
Immunodetection of calbindin in the inferior olive of 3-AP ataxic rats. (a) and (c) show calbindin immunostaining in two different levels of the inferior olive of 3-AP-lesioned rats separated by a distance of approximately 800 *μ*m. Note how HuUCBMCs grafts increase calbindin-positive cells in both zones of the structure (b, c). Scale bar: 150 *μ*m.

**Figure 4:**
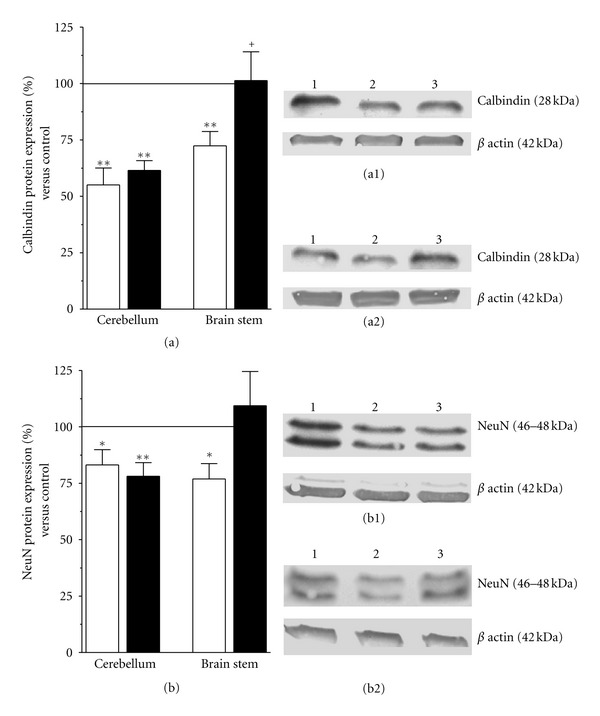
HuUCBMCs grafts partially prevent 3-AP-induced neuronal loss in the brain stem. (a) and (b) show the detection by western blot of calbindin (a) and neuronal nuclei (b, NeuN) in the cerebellum and brain stem of 3-AP-lesioned rats receiving vehicle (3-AP + vehicle rats, white bars) or HuUCBMCs grafts (3-AP + cells rats, black bars). (a1) and (a2) Show representative blots for calbindin, and (b1) and (b2) for NeuN in the cerebellum (a1, b1) and brain stem (a2, b2). Lane 1: naïve rats (control); lane 2: 3-AP + vehicle; lane 3: 3-AP + cells. Results represent the mean ± SEM of 6 to 9 individual animals. *P ≤ 0.05, **P ≤ 0.01 versus naïve rats, ^+^ *P* ≤ 0.05 versus 3-AP + vehicle rats.

A single injection of 40 mg/kg 3-AP significantly (*P* ≤ 0.001, *n* = 6) reduced the number of calbindin-positive neurons in zone 3 (Z3) of the inferior olive from 616 ± 22 to 220 ± 51 calbindin-positive cells/section in naïve and 3-AP-lesioned rats, respectively. A similar effect was observed in zone 1 (Z1) where calbindin-positive cells were reduced by 1.7-fold in 3-AP-lesioned rats (*P* ≤ 0.01, *n* = 4). As shown in Figure 3, after 48 days after lesion calbindin immunoreactivity was slightly higher in both zones of the inferior olive of 3-AP-lesioned rats that received HuUCBMCs (Figures 3(b) and 3(d)), compared to 3-AP + vehicle-treated rats (Figures 3(a) and 3(c)). We also analyzed the expression of calbindin and NeuN, a nuclear antigen expressed by neurons, by western blot. In the brain stem of 3-AP + vehicle-treated rats calbindin (Figure 4(a)) and NeuN (Figure 4(b)) protein levels were significantly lower than those found in naïve and 3-AP + cells treated rats. The neurotoxin 3-AP also reduced calbindin and NeuN protein expression in the cerebellum, but HuUCBMCs transplantation was unable to recover the levels of both proteins in this structure (Figures 4(a) and 4(b)).

### 3.4. HuUCBMCs Grafts Modulate Glial Reactivity in 3-AP-Lesioned Rats

**Figure 5:**
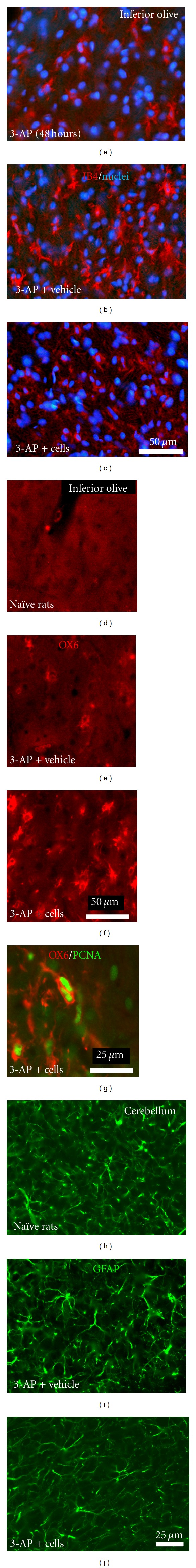
Immunodetection of glial cells in the inferior olive and cerebellum of 3-AP ataxic rats. (a) and (b) show the histochemical detection of isolectin B4 (IB4, red) in the inferior olive of 3-AP-lesioned rats 48 hours (a) and 10 days (b, c) after lesion. Note how 3-AP lesioned rats that received HuUCBMCs grafts (c, red) show lower IB4 staining at 10 days after lesion than ataxic rats treated with vehicle (b, red). (d) to (g) Show OX6 immunostaining in the inferior olive of naïve rats (d, red) and 3-AP-lesioned rats treated with vehicle (e, red) or HuUCBMCs (f, red). Cell transplantation increases OX6 immunoreactivity (f, red). (g) shows double immunolabeling for OX6 (red) and PCNA (green) in the inferior olive of 3-AP + cells-treated rats. (h) to (j) show GFAP immunoreactivity in the cerebellum. Note how HuUCBMCs grafts reduce GFAP immunolabeling in this structure (j, green). Nuclei were counterstained with Hoechst 33342 (blue). Scale bar: 25 *μ*m (g, h, I, and j), 50 *μ*m (a, b, c, d, e, and f).

**Figure 6:**
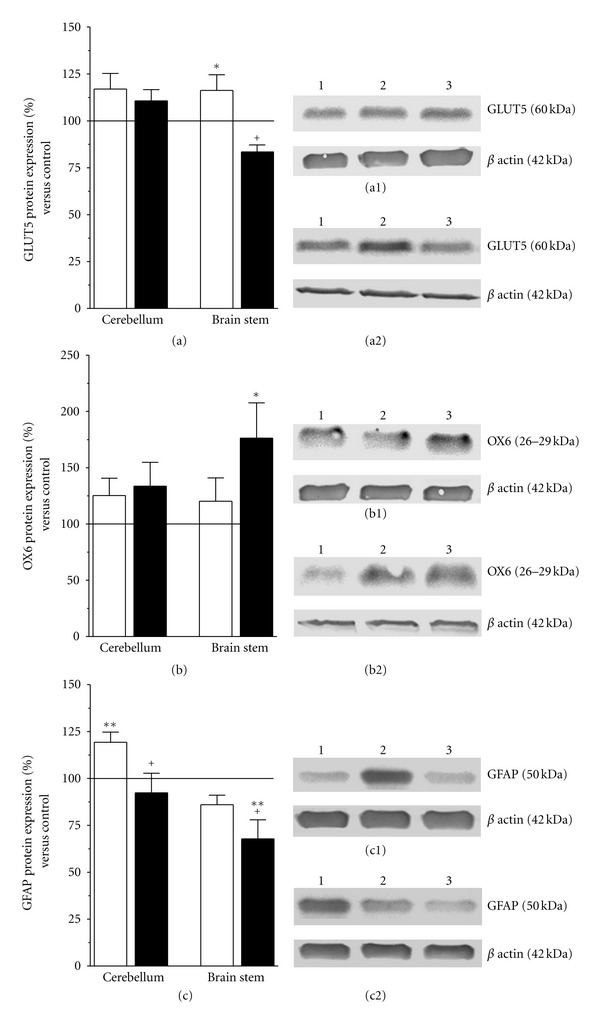
HuUCBMCs grafts modulate glial reactivity in 3-AP ataxic rats. (a), (b), and (c) show GLUT5 (a), OX6 (b), and GFAP (c) protein levels in the cerebellum and brain stem of 3-AP-lesioned rats receiving vehicle (3-AP + vehicle rats, white bars) or HuUCBMCs grafts (3-AP + cells rats, black bars). Note how HuUCBMCs transplantation upregulates OX6 protein expression in the brain stem and reduces GFAP protein levels in the cerebellum of 3-AP-lesioned rats. (a1) and (a2) show representative blots for GLUT5, (b1) and (b2) for OX6, and (c1) and (c2) for GFAP in the cerebellum (a1, b1, and c1) and brain stem (a2, b2, and c2). Lane 1: naïve rats (control); lane 2: 3-AP + vehicle; lane 3: 3-AP + cells. Results represent the mean ± SEM of 9 to 15 individual animals. *P ≤ 0.05, **P ≤ 0.01 versus naïve rats, ^+^ *P* ≤ 0.05 versus 3-AP + vehicle rats.

Previously, we observed that 3-AP induced a time-dependent invasion of cells expressing the vital marker of microglia IB4 in the inferior olive, that was maintained up to 24 days after lesion [46]. In agreement with those studies, the inferior olive of 3-AP + vehicle-treated rats showed a higher number of IB4-positive cells at 10 days after lesion than 48 hours after 3-AP was injected (Figures 5(a) and 5(b)). Moreover, IB4 labeling was increased in the inferior olive of 3-AP + vehicle-treated rats, as compared with 3-AP lesioned rats that received HuUCBMCs (Figures 5(b) and 5(c)). At 48 days after lesion, western blot analysis for the glucose transporter expressed by microglia GLUT5 gave similar results. Thus, GLUT5 protein expression was significantly raised in the brain stem of 3-AP + vehicle group of animals, compared to naïve and 3-AP + cells-treated rats (Figure 6(a)). 

The anti-OX6 antibody recognizes a histocompatiblility Class II antigen expressed by activated microglia. As shown in Figure 6(b), OX6 protein levels were raised by 1.76-fold in the brain stem of 3-AP + cell-treated rats. Similarly, OX6 immunoreactivity was increased in the inferior olive of 3-AP + cells rats (Figure 5(f)), compared to naïve (Figure 5(d)), and 3-AP + vehicle-treated animals (Figure 5(e)). Proliferation is another feature of microglial activation. HuUCBMCs transplantation upregulated PCNA protein expression by 1.6-fold in brain stem (**P ≤ 0.01 and ^+^ *P* ≤ 0.05 versus naïve and 3-AP + vehicle rats, resp.). In addition, some of the OX6-positive cells were PCNA positive in the inferior olive of 3-AP + cell-treated rats (Figure 5(g)). 

In the cerebellum, neither GLUT5 (Figure 6(a)), or OX6 (Figure 6(b)), nor PCNA protein levels were affected by HuUCBMCs transplantation, compared to naïve and 3-AP + vehicle-treated rats. However, the intravenous injection of HuUCBMCs prevented the increase in GFAP protein expression induced by 3-AP (Figure 6(c)). Besides, the cerebellum of 3-AP + cell-treated rats showed lower GFAP immunoreactivity than the cerebellum of 3-AP + vehicle rats (Figures 5(h)–5(j)).

### 3.5. HuUCBMCs Grafts Stimulate Bcl-2 Protein Expression and Phosphorylation of Akt

**Figure 7:**
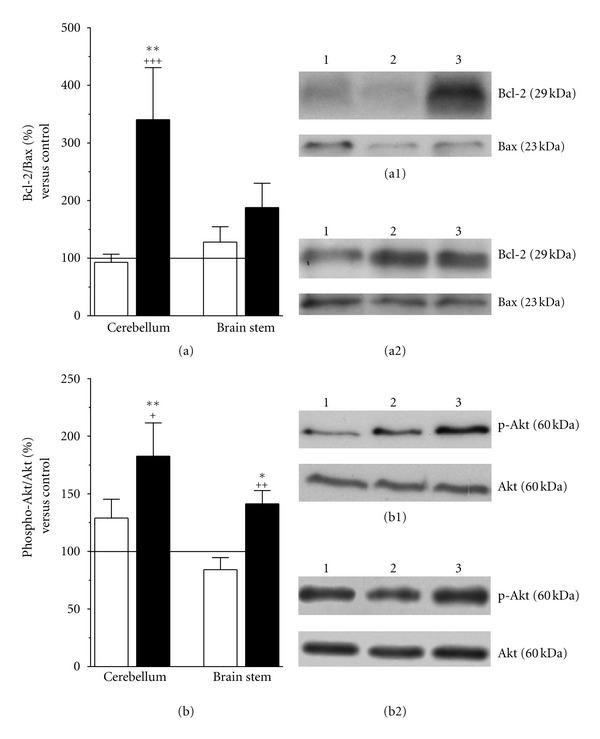
HuUCBMCs grafts upregulate Bcl-2 and phospho-Akt protein levels in the cerebellum and brain stem of 3-AP ataxic rats. HuUCBMCs transplantation raises the ratio Bcl2/Bax in the cerebellum of 3-AP-lesioned rats (a, black bars). Note how this effect is due to an increase in the expression of the antiapoptotic protein Bcl-2 (a1). HuUCBMCs also upregulate the ratio phospho-Akt/Akt in the cerebellum and brain stem of 3-AP ataxic rats (b, black bars). In both structures cell transplantation significantly enhances phospho-Akt levels (b1, b2), which is a protein involved in neuronal survival. (a1) and (a2) show representative blots for Bcl-2 and Bax, and (b1) and (b2) for phospho-Akt and Akt in the cerebellum (a1, b1) and brain stem (a2, b2). Lane 1: naïve rats (control); lane 2: 3-AP-lesioned rats treated with vehicle (3-AP + vehicle); lane 3: 3-AP-lesioned rats treated with HuUCBMCs grafts (3-AP + cells). Results represent the mean ± SEM of 9 to 15 individual animals. *P ≤ 0.05, **P ≤ 0.01 versus naïve rats, ^+^ *P* ≤ 0.05, ^++^ *P* ≤ 0.01, ^+++^ *P* ≤ 0.001 versus 3-AP + vehicle rats.

Several studies have proposed a neuroprotective role for HuUCBMCs [23]. Using western blot analysis, we studied the effects of HuUCBMCs grafts in the expression of proteins involved in cell survival. The Bcl-2 family comprise proteins that have either antiapoptotic (such as Bcl-2), or proapoptotic (such as Bax) effects [47–49]. In the cerebellum of 3-AP + cell-treated rats, the ratio Bcl-2/Bax was significantly raised compared to naïve rats and 3-AP + vehicle-treated animals (Figure 7(a)). This effect was due to the increase by 1.6-fold observed in Bcl-2 protein levels (*P ≤ 0.05 and ^+^ *P* ≤ 0.05 versus naïve and 3-AP + vehicle rats, resp.), while Bax levels remained unchanged in all experimental conditions studied (Figure 7(a1)). Neither 3-AP + vehicle rats, nor 3-AP + cell-treated animals showed significant changes in Bcl-2 and Bax protein expression in the brain stem (Figure 7(a)). 

The protein Akt is a key downstream effector of the PI3K/Akt-signaling pathway which phosphorylation plays a critical role in the regulation of neuronal survival [24, 50–54]. As shown in Figure 7(b), the ratio phospho-Akt/Akt was significantly increased in the cerebellum and brain stem of 3-AP + cells rats. HuUCBMCs grafts did not modify total Akt protein expression in both structures, but upregulated phospho-Akt levels in the cerebellum and brain stem of 3-AP-lesioned rats by 2- and 1.4-fold, respectively (*P < 0.05 versus naïve cerebellum and ^+^ *P* < 0.05 versus 3-AP + vehicle brain stem).

## 4. Discussion

In the present study we show that intravenous administered HuUCBMCs were able to reach the cerebellum and brain stem of 3-AP-lesioned rats. Implanted cells partially blocked the loss of neurons induced by the neurotoxin in the brain stem, prevented the overexpression of GFAP in cerebellum, and stimulated the expression of proteins involved in cell survival in both structures. All these effects were accompanied by a temporal but significant improvement in motor coordination, suggesting the potentiality of HuUCBMCs grafts as a cell replacement therapy for cerebellar disorders.

HuUCBMCs are considered an excellent source of stem cells that can be used for cell replacement therapies in neurodegeneration [20, 22, 23]. However, to our knowledge there are only two studies using HuUCBCs for the treatment of CA [39, 40]. By using an anti-HLA-ABC antibody, we found that intravenous administered HuUCBMCs reached the cerebellum and brain stem of 3-AP-lesioned rats. Under our experimental conditions, HLA-ABC immunostaining was only observed during the first three weeks of transplantation. However, western blot analysis showed HuNu protein expression in the cerebellum and brain stem of 3-AP + cell-treated rats two months after the administration of HuUCBMCs. These apparently contradictory results could be explained by a potential reduction in the expression of HLA-ABC at two months of transplantation.

In vitro and in vivo studies have demonstrated that HuUCBMCs are able to differentiate into neurons [31, 34, 43, 55, 56]. In ataxic rats HuUCBMCs implantation slightly recovered calbindin-positive neurons from 3-AP neurotoxicity in the inferior olive and restored calbindin and NeuN levels in brain stem. Although we found HLA-ABC-positive cells in the brain stem of 3-AP + cells-treated rats, none of these cells were located in the inferior olive. For this reason, we may infer that HuUCBMCs did not differentiate in calbindin-positive neurons in this structure. However, from our studies we cannot exclude the possibility that HuUCBMCs are able to differentiate in neurons in the brain stem as we were unable to detect HLA-ABC-positive cells after one month of transplantation. 

Increasing evidence strengthens the hypothesis that the beneficial role of transplanted HuUCBMCs is associated with the production of neuroprotective factors [14, 33, 36, 40, 57]. We did not analyze the expression of neurotrophins or cytokines and chemokines with anti-inflammatory properties, but we found that HuUCBMCs grafts potentiated the activation of microglia in the inferior olive and the brain stem of 3-AP-lesioned rats, as analyzed by OX6 and PCNA immunohistochemistry and immunoblot. Although activated microglia have been associated with the pathogenesis of several neurodegenerative diseases [58, 59], these cells could play a key role in neuroprotection through the production and release of neurotrophic factors [60, 61]. A recent study associated the therapeutic benefits of HuUCBMCs transplantation in a rat model of neonatal hypoxia with a transient up-regulation of microglial activity [62]. By contrast, the blockage of microglia activation enhanced neuroprotection and functional recovery induced by HuUCBMCs grafts in cortical ischemia [63]. Whether HuUCBMCs-driven activated microglia mediates the up-regulation of neuronal markers in the brain stem of 3-AP-lesioned rats or not, will be analyzed in the near future by blocking microglia activation with agents such as minocycline.

Up-regulation of GFAP is a feature of reactive astrocytes that was reported in the cerebellum of ataxic rats [64, 65], and patients suffering from progressive ataxia [66]. In agreement with those studies, we found increased GFAP protein levels in the cerebellum of 3-AP ataxic rats. This overexpression of GFAP could be the consequence of glial activation induced by a loss of neurons, as has been reported in several diseases and neuropathologies [67–69]. Although we did not analyze the number of neurons in the cerebellum, we found that 3-AP significantly decreased the expression of the neuronal markers calbindin and NeuN.

Rat umbilical cord stem cells grafts prevented reactive astrogliosis and rendered neuronal protection in the hyppocampus of ischemic rats [70]. In our study, HuUCBMCs transplantation reduced GFAP protein levels and immunoreactivity, but was unable to prevent the fall in calbindin and NeuN levels due to 3-AP neurotoxicity in the cerebellum. These results suggest that grafted cells could be involved in the modulation of astrogliosis, but they are not enough efficient to prevent neuronal damage in the cerebellum of 3-AP-ataxic rats.

An interesting finding was that HuUCBMCs transplantation modulated the expression of proteins involved in cell survival. As shown here, intravenous administration of HuUCBMCs significantly raised the phospho-Akt/Akt ratio in the cerebellum and brain stem of 3-AP + cell-treated rats. This effect was due to an increase in phospho-Akt levels, while total Akt remained unchanged in both structures. As other studies have shown that phospho-Akt plays a critical role in the regulation of neuronal survival induced by HuUCBMCs [71, 72], we may consider that this protein could mediate the neuroprotective activity of HuUCBMCs grafts observed in the brain stem. Phospho-Akt also contributes to glial cells survival [72–74]. However, there are no reports showing its possible role in preventing astrogliosis to our knowledge. The PI3K/Akt-signaling pathway regulates the expression of the antiapoptotic factor Bcl-2 [69, 75–77]. Here we found that HuUCBMCs grafts upregulated Bcl-2 protein expression in the cerebellum of 3-AP-lesioned rats. Bcl-2 overexpression enhanced the survival of different types of neurons [69, 77, 78], including those of the granular layer of the cerebellum [79]. Additionally, Bcl-2 may contribute to the maintenance of grafted cells in the cerebellum of 3-AP ataxic rats. In fact, Bcl-2 overexpression mediated the survival of human hematopoietic precursors during fetal life [80] and prolonged the survival of myoblasts transplantation in acute myocardial infarction [81] and chronic heart failure [82]. 

Finally, our results show that HuUCBMCs implantation ameliorates motor coordination in 3-AP-lesioned rat. This beneficial effect was probably due to neuronal protection elicited by the graft in the brain stem. In addition, HuUCBMCs could modulate neuronal activity in ataxic rats. In this respect, our preliminary studies have detected increased levels of glutamate and GABA in the brain stem of 3-AP-lesioned rats that were significantly reduced to basal levels in those animals receiving HuUCBMCs grafts (our unpublished observations). Under our experimental conditions, functional improvement was not maintained up to one month after transplantation. Recent studies have reported that repeated injections of HuUCBCs improved motor skills in ataxic mice and functional symptoms in patients with hereditary ataxia for longer periods of time [39, 40]. We administered a single intravenous injection of 4.5 × 10^6^ cells/rat, so further experiments are needed to determine the effectiveness of the repeated application of HuUCBMCs in the 3-AP-experimental model of cerebellar ataxia.

## 5. Conclusions

In summary, our results show that intravenous administered HuUCBMCs reach the cerebellum and brain stem of 3-AP-lesioned rats. Implanted cells stimulate the expression of proteins involved in cell survival in both structures. These proteins could mediate the survival of grafted cells, and the neuroprotective effect observed in the brain stem of ataxic rats. As HuUCBMCs grafts also ameliorate motor coordination in 3-AP-lesioned rats, they can be considered as a potential source of cells useful for cerebellar disorders treatment.
